# G-Protein Coupled Receptors (GPCRs): Signaling Pathways, Characterization, and Functions in Insect Physiology and Toxicology

**DOI:** 10.3390/ijms22105260

**Published:** 2021-05-17

**Authors:** Nannan Liu, Yifan Wang, Ting Li, Xuechun Feng

**Affiliations:** 1 Department of Entomology and Plant Pathology, Auburn University, Auburn, AL 36849, USA; yzw0093@auburn.edu (Y.W.); tzl0001@auburn.edu (T.L.); 2 Department of Biology Sciences, University of California, San Diego, CA 92093, USA; xfeng@UCSD.EDU

**Keywords:** G-protein coupled receptor regulation pathway, GPCR downstream effectors, functional characterization, insect physiology, insecticide resistance, cell lines

## Abstract

G-protein-coupled receptors (GPCRs) are known to play central roles in the physiology of many organisms. Members of this seven α-helical transmembrane protein family transduce the extracellular signals and regulate intracellular second messengers through coupling to heterotrimeric G-proteins, adenylate cyclase, cAMPs, and protein kinases. As a result of the critical function of GPCRs in cell physiology and biochemistry, they not only play important roles in cell biology and the medicines used to treat a wide range of human diseases but also in insects’ physiological functions. Recent studies have revealed the expression and function of GPCRs in insecticide resistance, improving our understanding of the molecular complexes governing the development of insecticide resistance. This article focuses on the review of G-protein coupled receptor (GPCR) signaling pathways in insect physiology, including insects’ reproduction, growth and development, stress responses, feeding, behaviors, and other physiological processes. Hormones and polypeptides that are involved in insect GPCR regulatory pathways are reviewed. The review also gives a brief introduction of GPCR pathways in organisms in general. At the end of the review, it provides the recent studies on the function of GPCRs in the development of insecticide resistance, focusing in particular on our current knowledge of the expression and function of GPCRs and their downstream regulation pathways and their roles in insecticide resistance and the regulation of resistance P450 gene expression. The latest insights into the exciting technological advances and new techniques for gene expression and functional characterization of the GPCRs in insects are provided.

## 1. Introduction

G-protein-coupled receptors (GPCRs), which are proteins sharing a seven α-helical transmembrane structure, govern a number of physiological processes in both the vertebrate and invertebrate kingdoms. The main function of GPCRs is to transduce extracellular signals and regulate intracellular second messengers through coupling to heterotrimeric G-proteins and their downstream effectors. As a result of the critical functions of GPCRs in cell physiology and biochemistry, they are important targets for the development of clinical medicines for a wide range of human disease therapies [1,2,3,4] and new and potentially more effective chemicals for insect pest management [5,6,7]. GPCRs are involved in recognizing extracellular messengers, transducing signals to the cytosol, and mediating the cellular responses necessary for the normal physiological functions of organisms [8,9,10,11,12,13]. GPCR binding to a wide variety of molecules (ligands) results in GPCRs interacting with G proteins that, in turn, activate the downstream effectors of GPCR pathways such as adenylate cyclase (AC), cyclic adenosine 3′,5′-monophosphate (cAMP), and protein kinase A (PKA), which regulate many different physiological processes [12,14,15,16,17,18]. GPCRs are particularly amenable to modulation by small-molecule drugs and therefore have been the targets of about half of the drugs currently used to treat humans [19,20]. Many exceptional studies have revealed how GPCR genes and their potential biological functions can impact insect physiology [21,22], including reproduction [23,24,25,26,27,28], regulating growth and development [27,29,30,31,32,33,34,35,36,37,38,39], the stress response [40,41,42,43,44,45,46,47,48,49,50,51], feeding [27,32,52,53,54,55,56,57,58,59,60], behaviors [23,61,62,63,64,65,66], and other physiological processes [46,67,68,69,70,71,72,73,74]. In addition, the overexpression of GPCRs in insecticide resistance has been reported in mosquitoes [49,50,51] and house flies [75]. In particular, the function of upregulated GPCRs and their downstream intracellular cascades has been investigated in the development of insecticide resistance in *Culex quinquefasciatus* [49,50,51]. All these exciting discoveries have combined to give us a good understanding of the complex molecular processes governing the development of insecticide resistance.

The last 10 years have seen a number of exciting technological advances and new techniques such as whole genome sequencing and high-throughput sequencing [76,77], double-stranded RNA-mediated gene interference (RNAi) [49,50,51], single nucleotide polymorphism determination [78,79,80], and cellular-based expression systems [81,82] are becoming widely available, enabling investigators to identify GPCR genes and leading to significant progress in characterizing the regulatory pathways in organisms, including insects. In this review article, we will mainly focus on the G-protein coupled receptor (GPCR) signaling pathways in insect physiology, including insects’ reproduction, growth and development, stress responses, feeding, behaviors, and other physiological processes. Hormones and polypeptides that are involved in insect GPCR regulatory pathways are reviewed.

We will give a brief introduction of GPCR pathways in organisms in general. At the end of the review, we will also provide a review of very recent studies on the function of GPCRs in the development of insecticide resistance, including our current knowledge of the expression and function of GPCRs and their downstream-regulation pathways in insecticide resistance through the regulation in resistance P450 gene expression. The latest insights into the exciting technological advances and new techniques for gene expression and functional characterization of the GPCRs in insects are provided.

## 2. GPCRs and the Regulatory Pathways Governing the Biological and Physiological Processes of Organisms

### 2.1. The GPCR Regulatory Pathways in General

**Figure 1:**
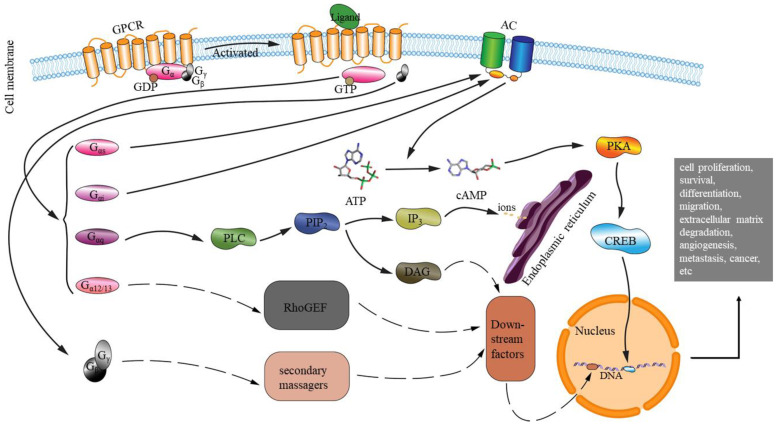
Graphical representation of the locations of GPCR regulatory pathways. GPCRs can be activated by a variety of ligands, interacting with heterotrimeric G-proteins composed of three subunits (Gα, Gβ, and Gγ) [95], and activating several downstream effector molecules [95]. Gα subunits are classified into four subfamilies: Gαs, Gαi, Gαq, and Gα_12/13_ [99,100] and can activate adenylyl cyclase (AC) [101], cyclic adenosine monophosphate (cAMP) [102,103,104], cAMP regulated proteins such as protein kinase A (PKA or cAMP-dependent protein kinase) [105], cyclic nucleotide-gated channels [106], and others [102,104,107], initiating and coordinating intracellular signaling pathways. Gαq can also activate phospholipase C (PLC), which can cleave phosphatidylinositol bisphosphate (PIP_2_) into diacylglycerol and inositol triphosphate (IP_3_) and membrane-bound diacylglycerol (DAG) [104,108,109,110]. IP3 can open the channel on the endoplasmic reticulum membrane [111], and DAG can activate protein kinase C [104,110]. Within the Gα_12/13_ family, G-α_13_ can increase the activation of p115RhoGEF (the Rho guanine nucleotide exchange factor) and related RhoGEF proteins linked to the Rho activation [112]. Several proteins are known to interact with Gα_12_, including Btk-family tyrosine kinase, Ras GTPase activating protein, cadherins, p120-caterin, and others [95,104,113,114,115,116,117]. Gβγ subunits can also send signals to phospholipase C, voltage gated Ca^2+^ channels, and others.

The signaling pathways of G-protein couple receptors and their downstream effectors that govern the cellular signaling transduction and physiological processes of organisms have been explored in the light of the huge amount of research data. The information generated have been greatly enhancing our understanding of the complex networks of GPCR pathways and how they affect living organisms’ biology and physiology. Researchers have examined insects [5,83,84], humans [85], nematodes [86], and other organisms [87,88], revealing that the GPCR regulation pathways play a crucial role in the cell signaling transduction that regulates important cell functions. These include cell proliferation [89], survival [89], differentiation [90], migration [91], extracellular matrix degradation [92], angiogenesis [93], metastasis [94], cancer [95], and others [95,96] (Figure 1). GPCR downstream effectors in many signaling pathways have been identified as potential targets by those working on developing new medicines for disease prevention and treatment in human populations. For example, in the phosphoinositide 3-kinase pathway, the efficacy of various inhibitors targeting downstream effectors, such as receptor tyrosine kinases, AKT, and mTOR, are now being tested in clinical trials [97]. The Hippo pathway present in both mammals and *Drosophila* and known to be involved in cell proliferation, death, and differentiation through regulating gene transcription is also being investigated [98].

### 2.2. GPCR Pathways and GPCR Downstream Effectors in Insect Physiology

**Figure 2:**
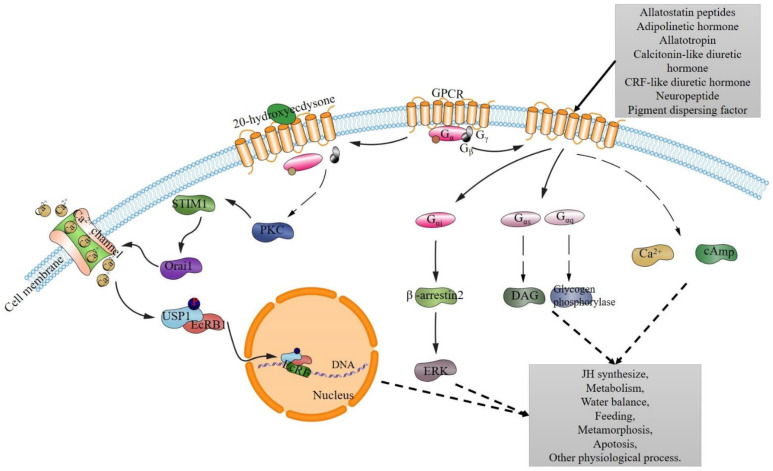
The proposed GPCR regulatory pathways in insect physiology processes.

A great deal of work is being done to characterize these hypothetical pathways and GPCR downstream effectors in insects [27,34,118,119,120,121]. Studies have revealed specific GPCR genes and their potential biological functions that may impact insects’ physiology [21,22], including their reproduction [23,24,25,26,27,28], and regulating their growth and development [27,29,30,31,32,33,34,35,36,37,38,39], as well as their stress responses [40,41,42,43,44,45,46,47,48,49,50,51], their feeding patterns [27,32,52,53,54,55,56,57,58,59,60], their other behaviors [23,61,62,63,64,65,66], and many other physiological processes [46,67,68,69,70,71,72,73,74]. A Moody-mediated signaling pathway has been identified that regulates the behavioral responses of *Drosophila* to cocaine and nicotine stimulation, suggesting a novel pathway could be involved in the functional changes observed in the blood–brain barrier that occurs in response to psychostimulants [64]. Several GPCRs are known to be triggered by neuropeptides, subsequently leading to intracellular pathways that are implicated in many physiological processes in insect species. For example, the adipokinetic hormone has been shown to bind with neuropeptide GPCRs to activate different G-protein subunits that regulate diverse signaling pathways, including DAG production and the activation of triacylglycerol lipase and glycogen phosphorylase, among others [83]. Insects can also use 20-hydroxyecdysone (20-E) or neuropeptides to stimulate GPCR pathways, which in turn regulate the gene expression involved in insect physiological functions such as apoptosis and the metamorphosis of arthropods [32,47,122,123,124] (Figure 2). A-type allatostatin neuropeptides and receptors have been discovered in juvenile hormone biosynthesis in many insect species, including drosophila, cockroaches, crickets, and termites [83], and an orphan neuropeptide receptor has been implicated in the silkworm’s overexpression of this GPCR in the corpora cardiaca and shown to be involved in the regulation of JH biosynthesis in the corpora allata [125]. Calcitonin-like diuretic hormones play a crucial role in the insect’s secretion of Malpighian tubules via a Ca^2+^-dependent mechanism and cAMP driven in GPCR regulation pathways [126,127]. In the Asian tiger mosquito, abundant GPCRs showed differential expression in blood feeding and non-blood feeding mosquitoes, indicating that the GPCRs could be involved in regulating multiple physiological pathways in the mosquito’s Malpighian tubules [128].

20-Hydroxyecdysone interacts with GPCR to initiate 20-E signal pathway [32,47,124] and triggers protein kinase C (PKC) [124]. PKC promotes the phosphorylation of the stromal interaction molecule 1 (STIM1), causing the aggregation of STIM1 [122]. The STIM1 interacts with the calcium release-activated calcium channel protein 1 (Orai1) to induce an influx of calcium ions [47], which stimulates the phosphorylation of ubiquitin specific peptidase 1 (USP1) and forms the USP1-EcRB1 (Ecdysone receptor) complex [32,47,122], which is then transferred to the nucleus and binds to the EcRE (ecdysone response elements) site to upregulate the genes involved in the various physiological functions [32,47,122,123]. Other neuropeptides and hormones also act as the ligands acting on the GPCR pathways, such as type C allatostatin peptide, which activates AST-C receptor to activate human Gαi protein and turn to activate β-arrestin2, and stimulate the activity of ERK finally [129]; adipokinetic hormone can bind with GPCR and activate Gα subunits to activate the glycogen phosphorylase and DAG [83,130]; and others can also bind with GPCR to process their physiological function through the cAMP or Ca^2+^ channel activation [67,83,126,131,132,133,134,135].

## 3. Gene Expression and Functional Characterization of the GPCRs in Insects

### 3.1. Whole Genome Sequence and Transcriptome Screening to Characterize the Genes and Efforts in the GPCR Regulatory Pathways

Over the last two decades, whole genome analysis and transcriptome gene expression have revolutionized efforts to identify and annotate insect GPCRs, and their potential functions can now be predicted using comparisons with other animal species. Indeed, the whole genome sequence and RNA-seq technologies have facilitated the research on GPCRs and their intracellular pathway studies and provided a large screening of GPCRs and their potential functions in insect physiology. In an early example demonstrating the power of these new technologies, a homology-based bioinformatics analysis conducted on the genome of the mosquito *Anopheles gambiae* revealed 276 GPCR gene annotations and several new classes [136]. The genome of the blowfly, *Lucilia cuprina*, has also been characterized by typical genomic sequencing, RNA-seq, and the assembly method. The subsequent genome sequence and gene/protein identification and annotation revealed 197 GPCRs and 167 kinases [137]. Several GPCRs and G-proteins were explored via reannotation in the fire ant, *Solenopsis invicta*, which combined with transcriptomic analyses made it possible to compare changes in the gene expression in the brain tissue of workers, alate virgin queens, and mated queens, revealing 10 GPCR and two G-protein genes with significantly different levels of expression [138]. In other recent work, a transcriptome study on GPCRs in the stick insect, *Carausius morosus*, conducted via RNAseq and de novo RNA assembly revealed 430 putative GPCR genes, some of which showed significant expression variations in different tissues [139]. Piermarini’s group employed de novo transcriptome assembly and analysis to identify the genes involved in blood feeding in the Asian tiger mosquito, *Aedes albopictus*; the subsequent differential analysis identified several GPCRs and metabolic genes that were upregulated or downregulated in the Malpighian tubules of blood-taken mosquitoes [128]. The availability of genome data has also sped up the research in insect toxicology and insecticide resistance. With the whole genome sequence data in mosquitoes *Cx. quinquefasciatus* [76], several GPCRs have been studied to characterize the functions in insecticide resistance in mosquitoes (Section 4). Scott et al. [77] have sequenced and analyzed the genome of the house fly *Musca domestica*, the vector of human and animal diseases, providing a rich resource for exploring the basic biology of this important pest, working on insect control, and understanding the mechanisms of insecticide resistance. Ninety-four putative G protein-coupled receptors (GPCRs) were identified in the *M. domestica* genome according to their homologues in *Drosophila melanogaster* [140]. The house fly genome information has provided valuable information for researchers to characterize the function of GPCRs in insecticide resistance (Section 4).

In addition to whole genome sequencing, microarrays are another effective way to characterize the differential expression of GPCRs in insects. For example, a microarray was utilized to determine the involvement of genes in the diapause process in the two-spotted spider mite, *Tetranychus urticae*, and reveal 916 upregulated and 1078 downregulated genes in diapausing females, of which four upregulated/downregulated genes were GPCRs [42]. In addition, a GPCR gene was identified from 1500 cDNAs showing upregulation in an insecticide-resistant *Culex* mosquito strain using subtractive PCR hybridization and cDNA microarray techniques [141].

### 3.2. Quantitative Gene Expression and Single Nucleotide Polymorphism Analyses of GPCR Genes in Insects

To better understand GPCR-specific expression and its potential involvement in physiological pathways, gene expression differences can be characterized in insects using quantitative expression methodology. For example, the expression of neuropeptide AKH and CRZ receptor transcripts have been characterized in the developmental stages and organs of adult *Aedes aegypti* mosquitoes [81], and a neuropeptide F-like receptor cDNA was cloned from *S. invicta* using PCR and RACE methods and the differential expression levels compared in the fire ant’s brain, guts, and reproductive systems, suggesting their potential involvement in the feeding regulation of mated queens [53]. A pheromone biosynthesis-activating neuropeptide (PBAN) and diapause hormone (DH) receptor were identified in the *Aedes* mosquito; screening the gene expression of PBAN-R and DH-R through the mosquito life stages suggested that although PBAN-R was downregulated in the late larval and pupal stages, the opposite was seen in the DH-R expression [46]. In addition, upon comparison of the gene expression of 115 GPCR and GPCR-related genes in insecticide susceptible and resistant strains of the mosquito, *Cx. quinquefasciatus* revealed four upregulated genes in the resistant strains [49] (Section 4.1).

Single nucleotide polymorphism (SNP) is another new technology that is being used to detect the nucleotide mutations of genes. In GPCR studies, it is utilized to identify the mutant receptors and the mechanisms involved. For example, it has been used to identify the mode of action in Amitraz, one of the formamidine acaricides widely used to control ticks. The involvement of the octopaminergic system and multiple mutations have been identified in the octopamine/tyramine receptor that are thought to contribute to Amitraz resistance in ticks [79]. PBAN also plays a critical role in the insect diapause process; PBAN receptors and protein kinase regulate the PBAN pathway in cells. Site-direct mutagenesis and protein structure prediction and modeling have also been utilized to determine the interaction sites for PBAN-R and the functionality of protein kinase in the corn earworm, *Helicoverpa zea* [80].

### 3.3. Functional Studies of GPCR Genes in Insects

RNAi is a robust biological tool with which to investigate the specific function of receptors in insects. Two putative crustacean cardioactive peptide receptors and their function were identified in the red flour beetle *Tribolium castaneum* using RNAi and heartbeat assay techniques, suggesting that one of them was indeed involved in the insect’s eclosion behavior and mediated its cardio acceleratory response [36]. An RNAi analysis also revealed that knockdown of the GPCR gene *AipsDopEcR*, which is overexpressed in the brain of a sexually mature male moth, *Agrotis ipsilon,* reduced the protein expression in the moth’s brain and inhibited its sexual behavior [63]. Similarly, the knockdown of one bursicon receptor (Tcrk) gene in *T. castaneum* in final instar larvae and a subsequent microarray and bioinformatics analysis revealed over a hundred differences in the gene expression between Tcrk RNAi-treated and control insects. These findings clearly identified the function of Tcrk in cuticle tanning of the insect pupa, ecdysis behavior, and adult wing and abdomen development [142]. A large-scale RNAi screen was also used to identify gene functions in insects. Injecting GPCRs-dsRNA into the larvae of red flour beetles caused mortality in 25 out of 111 GPCRs, with a further eight GPCRs affecting larval and pupal development [29]. Bai and Palli [24] went on to utilize RNAi to test the functions of 112 GPCRs in adult female *T. castaneum*, identifying two GPCR genes that are involved in vitellogenin uptake. A knockdown of BomNPFR in the silk moth *Bombyx mori* demonstrated its potential function in the moth’s food intake and growth processes [143]. Injecting a Corazonin receptor-dsRNA into the kissing bug, *Rhodnius prolixus*, and conducting a heartbeat assay confirmed its function in heartbeat control [144], while knockdown of a calcitonin receptor 1 gene in *Ae. aegypti* and measuring the subsequent midgut concentration identified its function in myotropic action in the female after a blood meal [70].

RNAi technology is not only being used to investigate the function of genes in vivo, it has also begun to be developed as a new type of insecticide for pest control known as Interfering RNA Pesticide (IRP). In *Ae. aegypti*, a TRP that targets GPCR-encoding dopamine 1 receptor (dop1) genes, has been shown to cause high mortality in adult mosquitoes [6]. Of course, this means that RNAi must now also be included in insecticide resistance studies. Studies of the knockdown resistance associated with GPCR/Gαs/AC/PKA pathway genes in insecticide-resistant mosquitoes have revealed increased susceptibility to insecticides (Section 4.2 and Section 4.3). Overexpression of the GPCR gene from the *Culex* mosquito in a *Drosophila* strain using transgenic technology showed increased resistance to insecticide and induced overexpression of P450s in the *Drosophila* [50]. A similar study in the gypsy moth *Lymantria dispar* that employed both RNAi and transgenic *Drosophila* techniques identified a methuselah-like GPCR that was functionally involved in the moth’s deltamethrin resistance and the regulation of its metabolic enzyme coding gene expression [145].

### 3.4. GPCR Characterization in Cell Line and Ligand-Binding Assays

To characterize the function of GPCRs and identify the agonists and antagonists to specific receptors, GPCRs are expressed in animal cells, and the intracellular messenger responses are monitored. Chinese hamster ovary (CHO) cells, human embryonic kidney (HEK293) cells, insect Sf9 and High 5 cells are commonly used to identify the GPCR functions. In a study to examine the ligand binding affinity, *Ae. aegypti* AKH and CRZ receptors were expressed in a recombinant CHO cell line, and a heterologous functional assay was conducted [81]. Similarly, two *Drosophila* putative tyramine receptors were stably expressed in CHO-K1 cell lines and agonist-dependent internalization was performed to identify the binding affinity of the ligands to their receptors. Then, the second messenger cAMP and Ca^2+^ levels were measured in the cell lines to confirm the intracellular activation of the tyramine receptors [146]. In other studies, four GPCRs from the mosquito, *Anopheles gambiae*, were transfected into CHO cells, and the reaction was tested with specific neuropeptides and biogenic amines to identify the GPCRs [147]. A short neuropeptide NPF receptor (sNPF) in *Drosophila* was characterized that binds with sNPF-like peptides involved in the insect’s food taking behavior and gene amplification, and a sequence comparison to the *Drosophila* genome was used to identify one putative GPCR gene, after which a bioluminescence assay was conducted in the CHO cell line to confirm that this gene does indeed encode the pyrokinin-1 receptor [148]. The intracellular regulatory mechanism of an adenosine receptor in the eclosion process was first identified in *Drosophila* by Dolezelova et al. [149]. In their study, the adenosine gene was stably expressed in the CHO cells, and subsequent treatment with adenosine revealed an increased concentration of second messengers. In the desert locust, *Schistocerca gregaria*, a functional study of a receptor expressed in the CHO and HEK cell lines utilizing ligand binding assays found that injecting peptide increased the insect’s food intake, confirming the regulatory pathway of sNPF in insects [54]. An octopamine receptor AmOctα2R in the honeybee that was transfected into HEK293 cells and monitored concentrations of Ca^2+^ and cAMP revealed that AmOctα2R was an octopamine receptor for tyramine [150]. A neuropeptide GPCR A4 receptor gene amplified from cells taken from a *B. mori* brain and transfected into a human embryonic kidney cell line (HEK93) and an insect *Spodoptera frugiperda* cell line (Sf9) increased the cAMP concentration, calcium flux, extracellular signal-regulated kinase activity, receptor location, and ligand-binding efficiency, revealing a GPCR-regulation pathway led by neuropeptide F that involved several intracellular factors, including Gαi, adenylyl cyclase, and Ca^2+^ flux [143].

A GPCR receptor corresponding to the PBAN/pyrokinin family of neuropeptides was first identified in the moth *Helocoverpa zea* as regulating pheromone production in an experiment that synthesized the cDNA of the PBAN receptor from the pheromone glands of female moths and then expressed it in insect *Sf9* cells in a ligand-binding assay to measure changes in the calcium-related fluorescence [46]. Then, a neuropeptide GPCR A19 isolated from *B. mori* and expressed in the HEK293T and sf9 cells was examined using confocal microscopy to detect the receptor expression and translocation in the cells. Treatment with inhibitors of Gq and PKC decreased the cell activities, indicating an intracellular pathway for the BNGR-A19/Gq/PKC/cAMP response to neuropeptide RYamides [151]. Several other receptors from various insect species have also been determined using cell expression systems. The characterization of a pyrokinin neuropeptide receptor in the Lyme disease vector, *Ixodes scapularis*, was conducted via gene isolation and expression in CHO cells [152]. An adipokinetic hormone receptor in the cockroach *Periplaneta* Americana was characterized in CHO/G-16 cells [153]. A leucokinin-like peptide receptor that was first identified in the Southern cattle tick, *Boophilus microplue*, was expressed and characterized via CHO-K1 cells [154].

An effective method that is widely used to identify the function of GPCR in insects is in vitro large-screening of selected compounds. For example, a *Drosophila* Methuselah receptor expressed in HEK-293 cells and its antagonist selection in 2800 natural products found that one of the natural insecticides, Rediocide A (1), inhibited the activation of GPCR by inducing conventional protein kinase C isoforms [155]. A putative tyramine receptor isolated from the southern cattle tick, *Rhipicephalus microplus*, and then expressed in CHO cells was found to exhibit differential responses to multiple compounds, revealing the pharmacologic importance of the receptor [156]. The cell-based screening of compounds to GPCRs to search for agonists and antagonists in *Aedes* mosquitoes is now being used to develop new insecticides [157]. Baculovirus-mediated infection is another option for studying GPCR functionality in vitro. In order to identify the structure of GPCRs, 16 human GPCRs were successfully expressed in *Sf9*, *Sf21*, and High Five insect cells using a recombinant baculovirus mediated expression system. Then, the membrane proteins collected were tested to determine their receptor activities and expression levels using radioligand-binding assays and immunoblotting [158]. The function of the GPCR-leading intracellular pathway in insecticide resistance has also been determined in insect *Sf9* cells [82,140] (Section 4.3).

### 3.5. Homology 3D Modeling Analyses of GPCRs

Since the first three-dimensional (3D) crystal structure of rhodopsin purified from bovine eyes was characterized in 2000 [159], tremendous progresses have made on understanding the structure–function relationships between ligand molecules and GPCR, which facilitated drug development at a remarkable speed [160]. As knowledge of interaction between GPCRs and their ligand molecules supports understanding their own biological and physiological functions, the research progress would reinforce the drug design. In addition, that knowledge would consequently achieve the developmental expansion of the new insecticides capable of interacting directly with GPCRs, even though none of insecticides have been designed in the light of direct target on GPCRs. Nevertheless, homology modeling technology has recently been used in insects to predict the 3D structure of a putative tyramine receptor in the rice weevil *Sitophilus oryzae*. Based on the predicted structure of the receptor, the ligand-binding affinity was employed to conduct a computer-based ligand selection as the initial step in the development of a new insecticide [161]. The discovery of GPCR and ligands in insects is now firmly established as a parallel study to human GPCRs. To identify a human vasopressin receptor and its antagonist, the oxytocin/vasopressin orthologue inotocin prepro-hormone sequences has been determined by transcriptome analysis from black garden ant, *Lasius nige*, workers, amplifying and expressing the inotocin receptor in CHO cells, and conducting radioligand binding assays, GPCR homology modeling, and in silico analysis [162]. Indeed, the variability of genome sequences of GPCRs in many insect species, the techniques for homology 3D modeling analyses, and availability of orthologue human GPCR protein structure and functions will facilitate the further research on insect GPCRs and their interacting ligand molecules, which may provide new information on developing new chemicals for pest control.

## 4. GPCRs, the Intracellular Pathway, and the Function into Insecticide Resistance

### 4.1. Overexpression of GPCR Genes in Insecticide Resistance

Insecticides, especially pyrethroids—the axonic neurotoxins acting on the voltage-sensitive sodium channels of the axonal membranes—are the most important weapon in our arsenal for the control of agriculturally, medically, and economically insect pests. However, the rapid development of resistance to insecticides is now becoming a serious issue in the insect pest control worldwide [163,164]. Insect cytochrome P450s are critical for the detoxification and/or activation of xenobiotics, including insecticides [164,165,166]. The overexpression of P450 genes, which leads to increased levels of P450 proteins and activities, resulting in increased metabolic detoxification of insecticides, including pyrethroids, has been implicated in the development of insecticide resistance of insects [164,165,166,167,168]. For example, several P450 genes, including *CYP6AA7* and *CYP9M10* [49], have been shown to be overexpressed in pyrethroid-resistant *Culex* mosquitoes [169]. Further characterization of the function of these overexpressed P450 genes revealed the association between changes in the expression levels of these overexpressed P450 genes and the levels of resistance to permethrin, one of pyrethroid insecticide, in mosquitoes, using RNAi and in silico modeling and docking analyses [49,170]. Using a baculovirus-mediated expression system and the enzymatic activity demonstrated the metabolic ability of *CYP9M10/CPR* and *CYP6AA7/CPR* to permethrin, confirming the important role played by *CYP9M10* and *CYP6AA7* in the detoxification of permethrin [171]. These significant findings highlight the functional importance of these P450 genes in insecticide resistance. However, cellular regulation pathways associated with the expression of these P450 genes are rarely identified. A first resistance-related GPCR gene was identified from 1500 cDNAs through the subtractive PCR and microarray techniques [141], showing upregulation in a pyrethroid-resistant *Cx. Quinquefasciatus* mosquito strain [141]. Subsequently, with the availability of the whole genome sequence for the mosquito *Cx. quinquefasciatus* [76], the expression levels of 115 GPCR and GPCR-related genes in the same insecticide-resistant *Culex* mosquitoes were characterized via the comparison with susceptible mosquitoes using quantitative real-time PCR. Four of *Culex* GPCR genes were identified as upregulated in the resistant strains, including the GPCR gene identified previously [141]. Consequently, several GPCR genes have been reported to be overexpressed in resistant insects, including mosquito *Culex pipiens pallens* [172], Asian gypsy moth, *L. dispar* [145], and house flies, *M. domestica* [140]. All these discoveries reveal the importance of the GPCRs involved in insecticide resistance of insects [49].

### 4.2. In Vitro Functional Studies of GPCR Regulation Pathways in Insecticide Resistance

**Figure 3:**
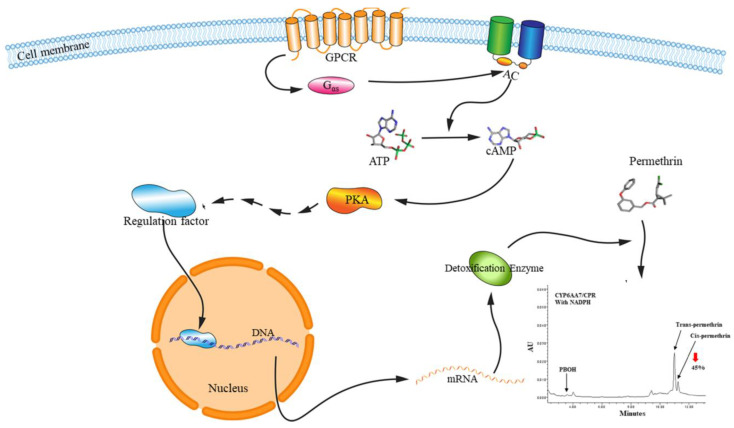
A hypothetical model of the G-protein-coupled receptor (GPCR) intracellular cascade in the insecticide resistance of insects, according to the hypothetical pathway constructed for GPCRs in human cells [95] and in mosquitoes [49,50,51]. The constitutive expressed GPCR in resistant mosquitoes activates the G-protein alpha s-subunit (Gαs), which stimulates adenylate cyclase to convert ATP to cAMP. cAMP activates the protein kinase A, which is involved in the increased expression of cytochrome P450 genes [49,50], resulting in elevating the detoxification ability of insects to insecticides. Inhibitions of cAMP production or PKA activity can interrupt this regulation pathway; the decreased production of cAMP or PKA activity is strongly associated with the decreased expression of resistance-related P450 genes and increased sensitivity to insecticides in both mosquitoes [49,50].

GPCR regulation pathways are known to play critical roles in insect physiology processes, and their involvement in the development of insecticide resistance is currently being explored. The first GPCR-leading intracellular pathway in insecticide resistance has been conducted in *Culex* mosquitoes [49,50,51,91,132]. These studies, using cAMP production inhibitor Bupivacaine HCl and the RNAi technique, for the first time revealed a GPCR regulatory pathway through Gαs, AC, PKA, and cAMP in mosquitoes that are involved in the regulation of resistance P450 gene expression, eventually leading to enhanced detoxification of insecticides by P450s and the development of resistance in *Culex* mosquitoes [49,50,51,96,173]. The function of the P450 genes, whose expression was thought to be regulated by this pathway, was also investigated via an RNAi-medicated expression system and a metabolic examination of permethrin, which is a pyrethroid insecticide, and its metabolites, revealing their robust capacity for insecticide metabolism [173] (Figure 3). The similar results have also been reported in *L. dispar* [145], in which RNA interference of a GPCR gene, *Ldmthl1*, resulted in a reduction of gypsy moths’ resistance to deltamethrin and the suppressed expression of P450 genes. The function of GPCRs in P450-mediated insecticide resistance could also been investigated through a *Drosophila* transgenic system. Li et al. [49] conducted a function study of rhodopsin-like GPCR using transgenic lines of *D. melanogaster*. The study found that not only the tolerance to permethrin insecticide increased in the transgenic lines of *D. melanogaster* was increased but also the expression of *Drosophila* resistance P450 genes, *CYP12d1* and *CYP6a8*, was increased [49], suggesting the involvement of GPCR genes in P450 gene expression and P450-mediated resistance. The similar functional studies using *Drosophila* transgenic lines have further confirmed the involvement of GPCRs in the regulation of P450 gene expression and insecticide resistance in *L. dispar* [145] and *M. domestica* [140].

### 4.3. In Vivo Functional Studies of GPCR Regulation Pathways in Insecticide Resistance

**Figure 4:**
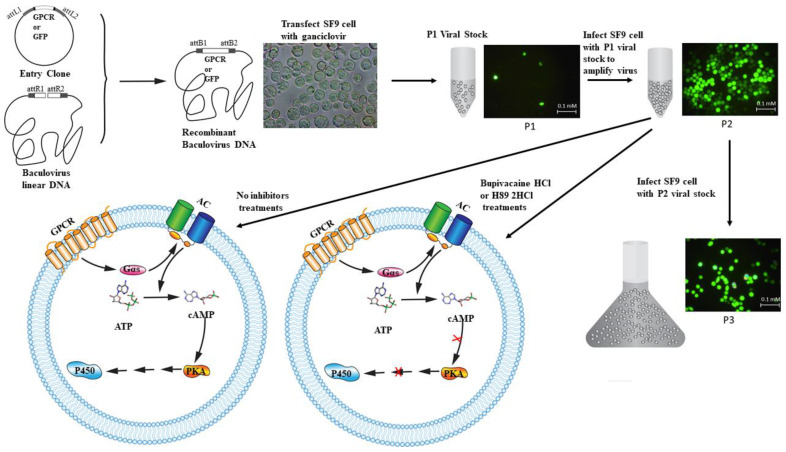
Functional study of GPCR-leading regulation pathway via recombinant baculovirus-GPCR or GFP expression in *Sf9* cells. A rhodopsin-like GPCR from the *Culex* mosquito or a GFP gene was constructed in pENTR^TM^ plasmid and then recombined with BaculoDirect Linear DNA to form a recombinant baculovirus of GPCR or GFP, expressed in insect *Sf9* cells. The recombinant baculovirus gene in the Phase 1 (P1) stage serves as the stock solution for virus amplification in Phase 2 (P2), in which GFP expression visually indicates that cells are alive and active, and thus suitable for use in gene functional studies [174]. The P2 virus continues to be amplified to Phase 3 (P3), which is retained as the final stock solution. A hypothesized GPCR-leading intracellular pathway has been determined for the recombinant virus-GPCR expression cells in P2. In these GPCR expression cells, PKA activity, cAMP production, and *Sf9* cell P450 gene expression can be examined after treatment with PKA activity inhibitor (H89 2HCl) or cAMP production inhibitor (Bupivacaine) for comparison with non-treated cells. Declining PKA activity and cAMP concentration, and the decreased expression of P450 genes confirm the involvement of the GPCR-leading pathway in insecticide resistance in vitro.

The function of the GPCR-leading intracellular pathway in insecticide resistance of Culex mosquitoes has also been evaluated and determined in a baculovirus-mediated expression system using *Spodoptera frugiperda* (Sf9) cells [82] to confirm the function of GPCR function and regulation pathway in vitro [96,173] (Section 4.2). In this study [82], a GPCR, Gαs, adenylate cyclase, protein kinase A gene from a *Culex* mosquito [173] was individually recombined with baculovirus expression in *Sf9* cells, and the PKA activity and cAMP concentration were measured, revealing significant increases in both. Interestingly, the expression of a few potentially resistance-related P450 genes in the *Sf9* cell increased, and an MTT assay with permethrin insecticide that was applied to the gene expression cells found higher survival ratios under insecticide treatment than in the control cells. Furthermore, a synergistic effect of a cAMP production inhibitor (Bupivacaine HCl) and PKA activity inhibitor (H89 2HCl) on the toxicity of permethrin resulted in decreased survival ratios in these cells, corresponding to increased concentrations of insecticide. Since the inhibitors may have a synergistic effect when used with the permethrin insecticide, these were then combined with permethrin to treat the *Culex* larvae, which showed increased susceptibility to the insecticides (Figure 4). All the factors were expressed in insect *Sf9* cells, which then showed increasing survival ratios in response to insecticide treatment, PKA activity, cAMP production, and *Sf9* cell native P450 gene expression [96,173]. As a robust gene functional expression system, successful baculovirus-mediated expression of insect GPCR genes has also been performed in *M. domestica* [140]. A cell-based toxicity assay demonstrated that the expression of a house fly GPCR gene, *LOC101899380*, could elevate the cell tolerance to imidacloprid insecticide [140].

## 5. Conclusions

**Figure 5:**
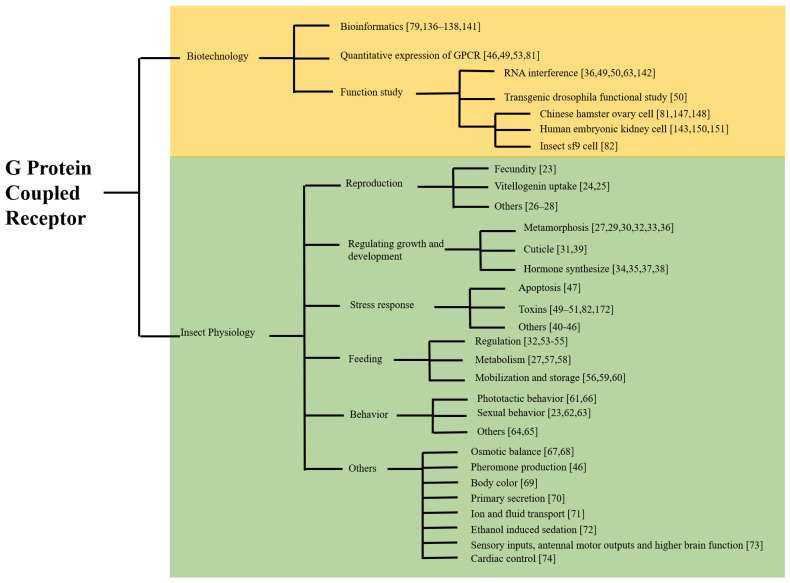
Graphical representation of the current status of GPCR research on insects and GPCRs and their regulatory pathways in insect physiology.

Scientists and researchers worldwide have been working diligently to elucidate the GPCR functions in cell biology, physiology, biochemistry, and molecular biology in both the vertebrate and invertebrate kingdoms. GPCRs are involvement in recognizing extracellular messengers, transducing signals to the cytosol, and mediating the cellular responses necessary for the normal physiological functionality of organisms. These functions are vital for targeting the development of novel therapeutic medications for a wide range of human diseases. GPCR research in insects has traveled a long way in just a few short years with a wide range of complementary technologies (Figure 5). It progresses from the initial GPCR gene identification to comprehensive bioinformatics analyses; from examining single mutations in a partial GPCR protein to characterizing multiple mutations over an entire target protein; from single GPCR gene analysis to whole GPCR pathway exploration; and from traditional transcriptional analysis of the gene expression to gene functional characterization. Insect GPCR research has revealed that GPCRs affect insects’ reproduction, growth and development, stress responses, feeding, behaviors, and other physiological processes (Figure 5). The research provides valuable information to guide the development of new insecticides for insect pest management. The outcomes of these studies provide us with a clear global picture that is enabling us to develop a clearer understanding of the highly complex mechanisms, genes, and pathways involved in the insect physiological processes. The insect GPCR research has also provides a strong foundation that will allow us to develop new insecticides and/or environmentally sound insecticides for better insect pest control. The findings reported in the most recent studies of GPCR functions in insects are opening up promising new avenues that will undoubtedly revolutionize future research on insect pest management.
